# Unmasking narcissistic leadership: a multilevel time-lagged study of the mediating role of employee resilience and the moderating role of supportive team climate

**DOI:** 10.3389/fpsyg.2026.1782797

**Published:** 2026-02-25

**Authors:** Xin Li, Juhee Hahn

**Affiliations:** 1 The Graduate School, Chung-Ang University, Seoul, Republic of Korea; 2 Department of Business Management, Chung-Ang University, Seoul, Republic of Korea

**Keywords:** employee resilience, employees’ innovative behavior, multilevel analysis, narcissistic leadership, supportive team climate

## Abstract

**Objectives:**

This study examines how narcissistic leadership influences employees’ innovative behavior in small and medium-sized enterprises (SMEs), focusing on employee resilience as a mediating mechanism and supportive team climate as a contextual moderator. Drawing on Conservation of Resources (COR) theory and Self-Determination Theory (SDT), the study clarifies the psychological and team-level processes through which narcissistic leadership affects innovation outcomes.

**Methodology:**

The study collected data through a three-wave, time-lagged survey from 445 employees nested within 84 teams in Chinese SMEs. To test the proposed cross-level mediation and moderation relationships, the study used multilevel structural equation modeling (MSEM).

**Findings:**

The results reveal a significant negative relationship between narcissistic leadership and employees’ innovative behaviors. Employee resilience mediates the relationship between narcissistic leadership and innovative behavior. In addition, a supportive team climate moderates the negative effect of narcissistic leadership on employees’ innovative behaviors, as well as the relationship between narcissistic leadership and employee resilience, such that these negative effects are weaker under conditions of high team-level support.

**Conclusion:**

These findings show that individual psychological resources and team-level contextual factors play critical roles in explaining how narcissistic leadership undermines employee innovation.

**Implications:**

This study contributes to the literature on destructive leadership by revealing how employee resilience and supportive team climate jointly shape innovation outcomes in multilevel organizational contexts. Practically, the findings suggest that cultivating supportive team environments can help buffer the harmful effects of narcissistic leadership by protecting employees’ psychological resilience and sustaining innovative engagement.

## 1 Introduction

In recent years, as organizations and leadership styles have evolved, narcissistic leadership has become more common in the corporate sector. This trend is particularly evident in rapidly growing small and medium-sized technology enterprises, where leader personality traits increasingly shape organizational operations amid accelerating socio-economic development (Shirokova et al., 2024). Although narcissistic leaders may occasionally enhance short-term performance due to their confidence and goal orientation, their excessive focus on personal interests and short-term outcomes often results in adverse consequences. This paradox suggests that apparent short-term gains may coexist with longer-term psychological and behavioral costs for employees, which become increasingly salient as leadership-related strain accumulates over time. Under such conditions, whether employees can sustain adaptive and innovative behaviors is likely to depend on the availability of contextual resources that buffer these longer-term negative effects. For instance, scholars have linked narcissistic leadership to corporate social irresponsibility and short-sighted managerial practices that undermine sustainable development and long-term performance (Grijalva and Harms, 2014; Agnihotri and Bhattacharya, 2021). Moreover, under conditions of uncertainty, narcissistic traits may distort organizational decision-making processes (Shirokova et al., 2023). Thus, examining narcissistic leadership in technology-oriented small and medium-sized enterprises (SMEs) is essential for understanding the organizational implications of dark-side leadership traits. Compared with large organizations, SMEs typically operate with less formalized structures and fewer resources, which increases employees’ dependence on leaders and limits their access to alternative sources of organizational support (Shirokova et al., 2024). Moreover, in the Chinese context, relatively high-power distance norms may further legitimize hierarchical authority, allowing self-centered leadership behaviors to exert more pervasive and enduring effects on employees’ psychological experiences.

To clarify these processes, this study draws primarily on the Conservation of Resources (COR) theory and integrates Self-Determination Theory (SDT) as a complementary lens to specify the motivational mechanisms through which narcissistic leadership affects employees’ psychological functioning and innovative behaviors (Braun et al., 2018; Norouzinik et al., 2022). From a motivational perspective consistent with SDT, narcissistic leaders’ controlling and self-centered behaviors are likely to frustrate employees’ psychological needs and undermine intrinsic motivations, which research shows impairs employees’ engagement in innovative activities (Braun et al., 2018; Norouzinik et al., 2022). COR theory explains how such sustained motivational strain contributes to resource depletion, thereby undermining employee resilience as a key personal resource for persisting in innovative effort (Hobfoll, 1989; Caniëls and Hatak, 2022; Chen et al., 2023).

Despite the growing prevalence of narcissistic leadership in technology-oriented SMEs, limited research has systematically examined how such leadership influences employees’ innovative behavior through underlying psychological mechanisms and under specific team-level contextual conditions (Braun et al., 2018; Norouzinik et al., 2022; Chen et al., 2023). Addressing this gap requires clarifying a mechanism that captures employees’ sustained capacity to adapt and continue innovating under chronic interpersonal strain, rather than only their momentary perceptions or task-specific beliefs. In this regard, employee resilience is particularly appropriate because it reflects a psychological resource that supports recovery and continued functioning when leadership-related stressors persist, which is highly relevant to innovation processes that involve uncertainty and repeated setbacks (Chen et al., 2023; Hobfoll, 1989). By contrast, constructs such as psychological safety or self-efficacy, while important, primarily emphasize situational appraisal or confidence in specific capabilities and may not fully capture the longer-term resource depletion and recovery dynamics triggered by narcissistic leadership (Hobfoll, 1989). This issue is particularly salient given the increasing reliance of SMEs on employee-driven innovation to sustain competitiveness in dynamic environments (Shirokova et al., 2024; Azeez, 2025).

As market competition intensifies and technological progress accelerates, organizations increasingly rely on employee innovation to respond to environmental changes. In technology firms, innovation is a key driver of competitive advantage and organizational agility. However, narcissistic leaders, characterized by egocentrism, excessive self-confidence, a strong need for recognition, and a desire for control (Fang et al., 2024; Khoso et al., 2025), often constrain employees’ creativity and autonomy (Fehn and Schütz, 2021). They often exclude employees from decision-making processes, thereby undermining employee motivation to engage in innovative behavior (Norouzinik et al., 2022). Accordingly, understanding the mechanisms through which narcissistic leadership influences employee innovation has become a critical topic in leadership research within SMEs.

Employee innovation refers to the process by which employees identify problems, generate novel ideas, and transform these ideas into practical applications (Paruzel et al., 2023). Prior research shows that factors at the individual, organizational, and interpersonal levels shape innovative behavior, including personality traits, work stress, skills, organizational culture, incentive systems, and leadership style (Jiang et al., 2023; Zhang et al., 2023; Zhu and Xie, 2023). Among these factors, leadership plays a particularly central role, as leaders directly shape employees’ psychological experiences and access to resources. However, compared with the extensive literature on positive leadership styles such as inclusive and humble leadership (Černe et al., 2013; Mostafa, 2023), the influence of dark-side leadership traits, particularly narcissistic leadership, on employee innovation remains relatively underexplored (Braun, 2017; Braun et al., 2018; Khoso et al., 2025).

Most existing studies on narcissistic leadership focus on individual traits and behavioral characteristics, such as confidence and power pursuit (Braun et al., 2018). Although recent research has begun to acknowledge the dual nature of narcissistic leadership (Eden et al., 2000; Faeq, 2025), its effects on employees’ psychological resources and innovation-related behaviors remain insufficiently understood. Moreover, although studies suggest that contextual factors such as supportive team climate buffer the negative effects of destructive leadership behaviors, scholars have rarely examined these influences within an integrated multilevel framework (Azam and Rizvi, 2021).

Research indicates that narcissistic leadership may undermine employee resilience, which we conceptualize here as a developable, context-sensitive psychological resource rather than a fixed trait, particularly in environments that lack supportive leadership (Caniëls and Hatak, 2022). The self-centered and controlling behaviors of narcissistic leaders can provoke emotional distress and deteriorate the work environment, thereby undermining employees’ psychological resources and triggering negative reactions (Mirzaei and Aghighi, 2024; Srivastava et al., 2025). Resilience serves as a critical psychological adaptation mechanism that enables employees to manage emotional challenges associated with narcissistic leadership and to sustain innovative behavior under pressure (Chen et al., 2023). Drawing on COR theory (Hobfoll, 1989), we can conceptualize employee resilience as a key personal resource that helps individuals withstand and recover from resource depletion caused by negative leadership. Supportive team environments likely strengthen this mediating process, where employees can access additional emotional and interpersonal resources that help buffer psychological threats and maintain engagement. Prior research further suggests that dark-side leadership traits, such as narcissism, often undermine trust and organizational identification, ultimately diminishing employee engagement and participation (Braun et al., 2018).

Despite these advances, several important research gaps remain. First, prior studies largely emphasize the direct effects of narcissistic leadership on employee outcomes, offering limited insight into the psychological mechanisms underlying its influence on employee innovative behavior (Braun et al., 2018; Norouzinik et al., 2022). Second, although scholars recognize employee resilience as a critical psychological resource in coping with workplace adversity, its mediating role in the context of narcissistic leadership remains insufficiently examined (Caniëls and Hatak, 2022; Mohammad et al., 2024; Srivastava et al., 2025). Third, existing research rarely incorporated team-level contextual factors, such as supportive team climate, into a multilevel framework to examine whether and how such contextual resources buffer the negative effects of narcissistic leadership (Azam and Rizvi, 2021; Zhou et al., 2019).

By addressing these gaps, this study makes several contributions. First, it advances research on narcissistic leadership by identifying employee resilience as a key mediating mechanism linking narcissistic leadership to innovative behavior. Second, it adopts a multilevel perspective by incorporating supportive team climate as a contextual moderator. Third, methodologically, this study employs a time-lagged design and multilevel structural equation modeling to examine cross-level relationships in SMEs.

Consequently, this study explores the following key research questions (RQ):

*RQ1*: How does narcissistic leadership influence employees’ innovative behaviors in small- and medium-sized enterprises?

*RQ2*: Does employee resilience mediate the relationship between narcissistic leadership and employees’ innovative behavior?

*RQ3*: Under what conditions does a supportive team climate mitigate the resource-depleting effects of narcissistic leadership on employee resilience and innovative behavior?

To address these research questions, this study first develops the theoretical framework and hypotheses based on relevant literature. It then outlines the research design and analytical procedures, followed by the presentation of empirical results. Finally, we discuss the implications of the findings, along with limitations and directions for future research.

**Figure 1:**
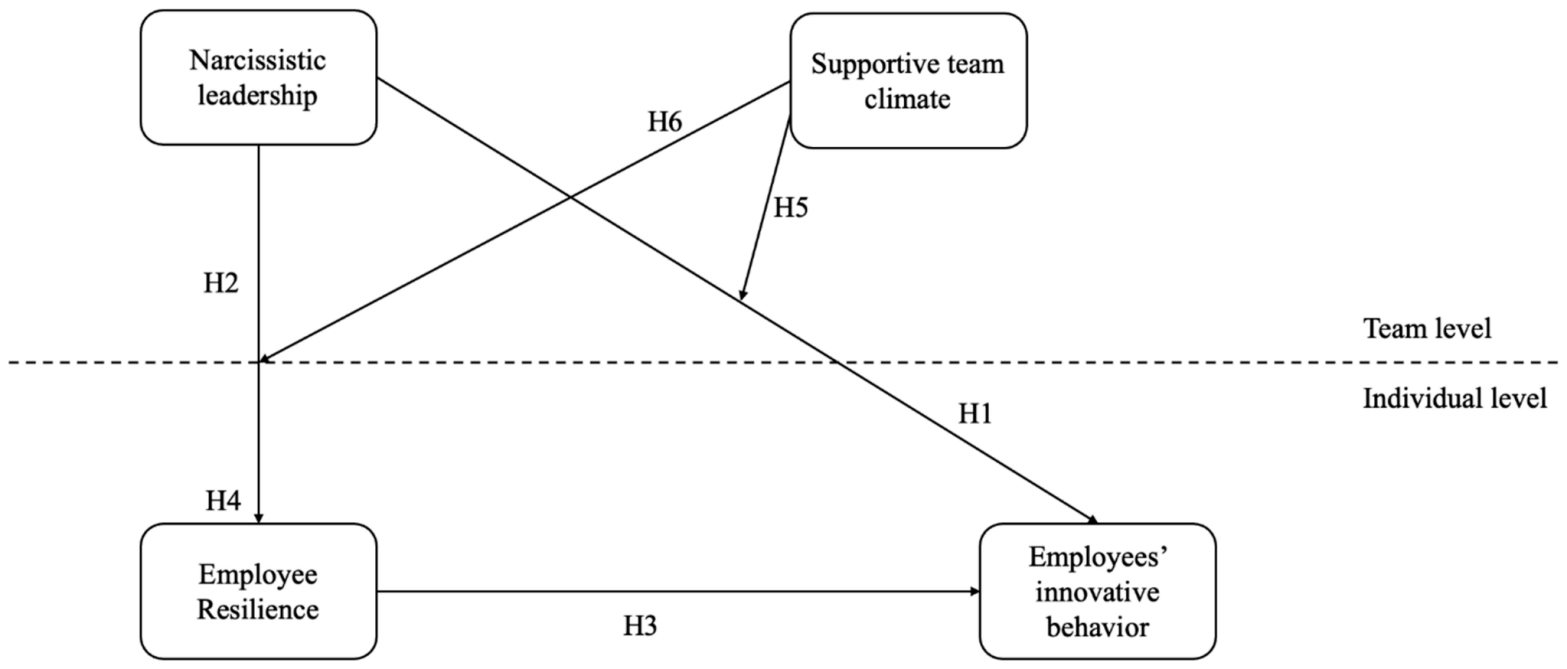
Research model.

Figure 1 presents this study’s research framework.

## 2 Theoretical background and hypothesis development

### 2.1 Narcissistic leadership and employees’ innovative behaviors

Narcissistic leaders exhibit heightened self-confidence, a marked egocentric orientation, a strong desire for power, and a significant need for recognition (Fang et al., 2024). Narcissistic chief executive officers (CEOs) anticipate that their bold actions will receive significant acclaim in instances of elevated audience engagement; therefore, they tend to invest aggressively in innovative technologies (Gerstner et al., 2013). Such leaders emphasize personal achievements, dismiss alternative viewpoints, and frequently disregard subordinates’ emotions and needs (Wang et al., 2022). At the interpersonal level, these characteristics shape how narcissistic leaders respond to employees’ innovative efforts. Fearing that employees’ innovative success might overshadow them, leaders may intentionally suppress those employees’ innovative efforts (Chatterjee and Hambrick, 2007). Narcissistic leaders frequently engage in manipulation and exploitation to advance self-interest, and they may claim credit for their subordinates’ innovative efforts (Locke et al., 2009). Recent studies further suggest a consistent association between narcissistic leadership and related toxic leadership patterns with adverse workplace experiences that undermine creativity and innovation-related performance (Khoso et al., 2025; Faeq, 2025).

Innovative behavior frequently entails rejecting existing work methods and processes, and this may embarrass narcissistic leaders; therefore, they may oppose innovation or even respond negatively to it (Yang et al., 2021; Huu, 2023). According to COR theory, engaging in innovative behavior requires substantial psychological resources, as it involves uncertainty, risk-taking, and sustained effort (Kiazad et al., 2014). From a resource-based perspective, narcissistic leadership, characterized by controlling, self-serving, and psychologically draining behaviors, accelerates employees’ resource depletion, thereby reducing their capacity and willingness to engage in innovative activities. Empirical studies indicate that narcissistic leaders often exaggerate their achievements, downplay subordinates’ contributions, and limit employees’ involvement in decision-making processes (Norouzinik et al., 2022), thereby undermining the perceived significance of innovation.

From a relational perspective, Leader–Member Exchange (LMX) theory, which focuses on the quality of the relationship between leaders and employees, helps explain how narcissistic leadership shapes day-to-day interpersonal experiences and further constrains employees’ access to psychological resources. Leaders may withdraw support, withhold benefits, and direct criticism or blame toward employees who do not demonstrate innovate behaviors. LMX theory posits that high-quality leader–member relationships enhance employees’ innovation motivations (Echebiri and Amundsen, 2021). However, narcissistic leaders are more likely to establish low-quality and unequal exchange relationships, which weaken employees’ sense of belonging and psychological safety, thereby exacerbating resource depletion and discouraging voice and experimentation (Mascareño et al., 2020). In the long run, although this leadership style may occasionally result in breakthrough innovations, it tends to suppress employees’ proactivity and creativity (Dworkis and Young, 2023). Consequently, employees may doubt the significance of innovation, lack the willingness and motivation to participate, and become increasingly unlikely to generate and implement innovative ideas in their roles. Therefore, this study proposes the following hypothesis:

*Hypothesis 1*: Narcissistic leadership negatively impacts employees’ innovative behaviors.

### 2.2 Narcissistic leadership and employee resilience

Employee resilience refers to an individual’s capacity to maintain a positive and adaptive state, recover quickly, and sustain effective performance when confronted with workplace stress, challenges, or adversity (Luthans et al., 2006). This study defines employee resilience as a dynamic and developable psychological resource that employees can strengthen or deplete depending on their ongoing work experiences and leadership contexts. It serves as a critical psychological resource at the individual level and a foundation for high performance, innovation, and commitment within the organization. According to COR theory, individuals endeavor to protect their limited psychological resources, such as confidence and positive emotions. When external stressors deplete these resources, employees are likely to experience psychological exhaustion and diminished adaptability (Kiazad et al., 2014). Leadership behavior that induces high uncertainty, characterized by excessive control or constant change, tends to heighten employees’ feelings of anxiety, stress, and helplessness. Such sustained strain undermines employees’ ability to replenish psychological resources, thereby weakening resilience as a recoverable capacity rather than a fixed attribute (Lyons and Schneider, 2009).

Narcissistic leadership poses a significant internal threat to employee resilience. At the organizational level, the dysfunctional characteristics of narcissistic leaders may inflict substantial psychological strain on employees, resulting in feelings of powerlessness and disengagement (Azeez, 2025). Recent research further indicates an association between narcissistic leaders’ ego-driven behaviors and low empathy and employees’ heightened emotional suppression and reduced psychological recovery (Srivastava et al., 2025). From a COR perspective, such ego-centered and controlling behaviors represent chronic resource-draining conditions that erode employees’ capacity to recover from stress and adapt to ongoing demands. These dynamics undermine available organizational and interpersonal support, thereby weakening employees’ adaptive functioning and psychological resilience. Although narcissistic leaders may exhibit psychological capital attributes such as confidence and charisma (Cragun et al., 2020), scholars emphasize that their arrogance, self-centeredness, and intolerance of criticism more often result in adverse outcomes, particularly the deterioration of employees’ psychological safety and recovery capabilities.

More recent evidence suggests that narcissistic leadership diminishes employees’ capacity for emotional regulation and self-recovery, thereby undermining resilience as a key psychological resource in demanding work contexts (Faeq, 2025). Furthermore, narcissistic leaders frequently prioritize personal agendas over employee development, thereby suppressing subordinates’ proactivity and self-regulatory capacity (Dworkis and Young, 2023). Taken together, these findings show that narcissistic leadership can systematically deplete employee resilience over time, highlighting resilience as a malleable psychological resource rather than a fixed trait.

Based on these theoretical and empirical insights, this study proposes the following hypothesis:

*Hypothesis 2*: Narcissistic leadership negatively impacts employee resilience.

### 2.3 Employee resilience and employees’ innovative behaviors

Employee resilience refers to an individual’s psychological capacity to preserve emotional stability, positively adapt when confronted with stress, challenges, or adversity, quickly recover from setbacks, and sustain effective goal-directed performance (Luthans et al., 2006). Within organizational contexts, resilience acts as a defensive buffer against occupational stress and a proactive psychological resource that fosters personal growth and transforms challenges into opportunities (Caniëls and Hatak, 2022). The COR theory posits that individuals need to acquire, preserve, and mobilize psychological resources to navigate high-intensity work demands and environmental uncertainty (Kiazad et al., 2014). Accordingly, we can understand resilience as a context-sensitive psychological resource that supports sustained engagement and flexible problem-solving under resource strain (Kiazad et al., 2014; Caniëls and Hatak, 2022). Previous research indicates that psychologically resilient employees tend to engage in proactive innovation, process enhancement, and ongoing experimentation (Qian et al., 2024).

Additionally, self-determination theory posits that the fulfillment of basic psychological needs, namely autonomy, competence, and relatedness, leads to enhanced intrinsic motivation and exploratory behavior among individuals (Deci and Ryan, 2012; Gagné and Deci, 2005). Employees with high resilience tend to exhibit greater self-efficacy and persistence in achieving their goals. This adaptive capacity helps them sustain intrinsic motivation and exploratory effort across innovation stages (e.g., idea generation, selection, and implementation) (Scott and Bruce, 1994; Deci and Ryan, 2012; Gagné and Deci, 2005). Furthermore, resilient individuals tend to uphold their exploratory intent and self-regulation, even in contexts marked by unsupportive leadership or limited organizational resources. This capacity helps to mitigate emotional exhaustion and strengthen organizational commitment. Supporting this perspective, Krasikova et al. (2013) discovered that highly resilient employees demonstrate enhanced recovery capacity and creativity when experiencing uncertainty or failure. Similarly, Mohammad et al. (2024) emphasized the essential function of resilience in enabling proactive engagement and adaptive performance within challenging work settings.

More recent evidence further reinforces this relationship by showing that employee resilience directly contributes to innovative performance, particularly in adverse or stressful workplace conditions. For example, Ullah et al. (2025) demonstrated that resilient employees are better able to buffer negative workplace experiences and sustain innovative performance, underscoring resilience as a key psychological mechanism linking adversity to innovation-related outcomes.

Collectively, these findings indicate that resilience functions as a moderating buffer, facilitating recovery from adversity and serves as a fundamental mediating mechanism linking psychological adaptability to innovative work behavior. Accordingly, this study proposes the following hypothesis:

*Hypothesis 3*: Employee resilience positively impacts employees’ innovative behaviors.

### 2.4 Mediating role of employee resilience in the relationship between narcissistic leadership and employees’ innovative behavior

Employee resilience plays a critical role in explaining how individuals psychologically adapt to adverse leadership contexts. In this study, resilience functions as an internal regulatory mechanism that links narcissistic leadership to employees’ innovative behaviors. Studies show that narcissistic leadership intensifies psychological strain and accelerates the depletion of employees’ psychological resources (Bauer et al., 2014; Wang et al., 2022). According to COR theory, when individuals experience sustained resource loss and lack opportunities for replenishment, their capacity to cope with stressors and engage in discretionary behaviors such as innovation diminishes substantially (Kiazad et al., 2014).

Employee resilience alters this resource depletion process by enabling individuals to regulate negative emotions, maintain psychological equilibrium, and preserve goal-directed focus under adverse conditions. Employees with higher resilience reinterpret stressful leadership experiences, buffer emotional exhaustion, and sustain adaptive functioning even in high-pressure environments (Sutcliffe and Vogus, 2003). From a motivational perspective grounded in self-determination theory, resilience helps employees maintain intrinsic motivation and a proactive orientation under controlling or unsupportive leadership conditions, enabling them to continue engaging in innovative activities despite constrained external resources (Mohammad et al., 2024).

Taken together, narcissistic leadership undermines employees’ psychological resources, whereas employee resilience counteracts this depletion by restoring adaptive capacity and sustaining innovative engagement. Thus, resilience serves as a key psychological pathway that transmits the resource-depleting effects of narcissistic leadership to employees’ innovative behaviors (Kiazad et al., 2014; Mohammad et al., 2024).

Based on this theoretical foundation, this study proposes the following hypothesis:

*Hypothesis 4*: Employee resilience mediates the relationship between narcissistic leadership and employees’ innovative behaviors.

### 2.5 Moderating effect of supportive team climate **i**n the relationship between narcissistic leadership and employees’ innovative behavior**s**

Recent research in organizational behavior suggests that the effects of leadership on employee innovation are not uniform but contingent on contextual conditions within the work environment. Among these conditions, a supportive team climate is a critical buffering mechanism that can shape employees’ behavioral responses to adverse leadership (Schneider et al., 2013). In this study, supportive team climate functions as a contextual resource that alters how employees interpret and respond to narcissistic leadership. In teams characterized by trust, collaboration, open communication, and emotional support, employees are more likely to perceive respect, inclusion, and shared responsibility, which collectively foster innovative engagement (Pasricha et al., 2023; Surianto and Nurfahira, 2024).

When leaders exhibit narcissistic tendencies, such as authority seeking, emotional neglect, and unilateral decision-making, employees often experience constrained self-expression and heightened psychological strain, which suppresses creativity and proactive behavior (Wang et al., 2022; Norouzinik et al., 2022). From a resource-based perspective grounded in COR theory, a supportive team climate provides alternative interpersonal and social resources that help employees regulate negative emotions and restore psychological balance (Zhou et al., 2019). This access to alternative support systems enhances employees’ sense of control and psychological safety, thereby weakening the adverse perceptions associated with narcissistic leadership.

Insights from situational strength theory and social information processing theory help explain why supportive team climate can attenuate the negative effects of narcissistic leadership. Situational strength theory posits that strong contextual cues reduce ambiguity in behavioral expectations and guide individual responses to external stimuli (Mischel, 1977), while social information processing theory suggests that individuals rely on environmental cues to interpret leadership behavior and determine appropriate responses (Salancik and Pfeffer, 1978). A supportive team climate provides salient social cues that shift employees’ attention away from leader-centric pressure toward shared goals and mutual support, thereby sustaining innovative motivation and implementation even under directive or authoritarian leadership conditions (Li and Liu, 2023).

Therefore, a supportive team climate functions as an alternative source of social support and a critical interpretive framework that reshapes employees’ cognitive and emotional reactions to narcissistic leadership. As a result, it can substantially attenuate the negative impact of narcissistic leadership on employees’ innovative behaviors. Based on these theoretical arguments, this study proposes the following hypothesis:

*Hypothesis 5*: A supportive team climate moderates the relationship between narcissistic leadership and employees’ innovative behaviors, such that a highly supportive team climate significantly reduces the negative impact of narcissistic leadership on those behaviors.

### 2.6 Moderating effect of supportive team climate in the relationship between narcissistic leadership and employee resilience

In today’s complex and dynamic organizations, employee resilience helps individuals adapt to challenges and recover from adversity. However, negative leadership does not erode or preserve resilience uniformly; instead, team-level contextual resources largely determine whether employees maintain or lose resilience. Prior research indicates that narcissistic leaders often manage teams in a self-centered manner characterized by excessive control, emotional neglect, resistance to criticism, and an overemphasis on authority (Braun, 2017; Cragun et al., 2020; Wang et al., 2022). Such leadership behaviors undermine employees’ autonomy and psychological safety, accelerate the depletion of psychological resources, and weaken coping capacity, thereby constraining the development and expression of employee resilience (Bauer et al., 2014; Norouzinik et al., 2022). These detrimental effects become especially pronounced when employees lack alternative sources of interpersonal and social support.

In this context, a supportive team climate serves as a critical contextual resource that can moderate the impact of narcissistic leadership on employee resilience. Consistent with COR theory, a supportive team climate provides alternative emotional and social resources that help offset leader-induced resource depletion (Rhoades and Eisenberger, 2002; Schneider et al., 2013). By enhancing employees’ sense of belonging and perceived support, supportive teams reduce employees’ sensitivity to leader-driven stressors and diminish the perceived threat associated with narcissistic leadership behaviors (Edmondson, 1999). Through ongoing peer interactions, supportive teams function as informal recovery systems that facilitate emotional regulation and resource replenishment, even in the presence of controlling or self-serving leaders (Li et al., 2024; Zhang et al., 2025).

Moreover, a supportive team climate provides stable social cues and consistent feedback that guide adaptive responses, enabling employees to maintain psychological balance and remain flexible in the face of uncertainty and pressure (Surianto and Nurfahira, 2024). Taken together, supportive team climate reshapes the contextual conditions under which narcissistic leadership influences employee resilience by buffering resource loss and facilitating recovery. As a result, a highly supportive team climate weakens the negative impact of narcissistic leadership on employee resilience. Based on this theoretical reasoning, this study proposes the following hypothesis:

*Hypothesis 6*: A supportive team climate moderates the relationship between narcissistic leadership and employee resilience, such that a highly supportive team climate significantly diminishes the negative impact of narcissistic leadership on employee resilience.

## 3 Methods

### 3.1 Sample and procedure

This study follows a deductive research logic and uses a quantitative, theory-testing design to examine the cross-level relationships among narcissistic leadership, employee resilience, and innovative behavior. The study tests theoretically grounded hypotheses, as well as mediation and moderation mechanisms, by employing a time-lagged multilevel structural equation model and gathering survey data from leaders and employees within fast-growing technology SMEs in China. The selected regions include Beijing, Shanghai, Guangzhou, Shenzhen, and Hangzhou. We first contacted the human resources departments of participating companies, which then helped us connect with team leaders after obtaining their consent. To ensure relevant experience, the study required all respondents to have worked at their current organization for at least 6 months (Sameer, 2018).

We administered the survey in three waves and assigned a unique code to each questionnaire to match team leaders with their team members across all three periods. In the first wave (September 13, 2024), we asked employees to assess narcissistic leadership and supportive team climate. In the second wave (October 7, 2024), employees completed a self-report measure of resilience. In the third and final wave (October 28, 2024), team leaders evaluated their subordinates’ innovative behaviors.

We designed the questionnaire to ensure semantic clarity and logical consistency. We administered all survey items in Chinese and used a rigorous back-translation procedure to preserve semantic equivalence because all participants were native Chinese speakers (Brislin and Freimanis, 2001). Additionally, we ensured strict confidentiality by using all collected data solely for this study and handling it anonymously and discreetly.

To support accurate evaluation and data matching, team leaders completed paper-based questionnaires to assess their subordinates’ innovative behaviors. We labeled each questionnaire with the corresponding team member’s name and code. After screening the data, we excluded 23 incomplete or invalid responses and retained 445 valid responses from 84 teams for analysis. These respondents represented a range of job functions, including technical, sales, and managerial roles.

This study used SPSS 29.0 to analyze the descriptive statistics of the respondents’ demographic characteristics. Among the 445 team members, 227 (51.0%) were female, and 218 (49.0%) were male.

Employees ranged in age from 20 to over 50 years old. Specifically, 11.0% were aged 20–30, 39.1% were 31–40, 33.5% were 41–50, and 16.4% were over 50 years old. In terms of education, 18.7% had completed college or less, 61.8% held a bachelor’s degree, and 19.6% held a master’s degree or higher. Regarding work experience, 2.0% had less than 2 years, 18.4% had 2–5 years, 36.4% had 6–10 years, and 43.1% had more than 10 years.

Among team leaders, 31 (36.9%) and 53 (63.1%) were male. Their age distribution was as follows: 4.8% were 20–30, 29.8% were 31–40, 48.8% were 41–50, and 16.7% were over 50. Regarding education, 4.8% had completed college or less, 70.2% held a bachelor’s degree, and 25.0% held a master’s degree or higher. In terms of experience, 17.9% had 2–5 years, 16.7% had 6–10 years, and 65.5% had over 10 years.

Team sizes also varied. Most teams had two-to-five members (84.5%), followed by teams with six-to-seven members (8.3%), eight-to-ten members (4.8%), and more than ten members (2.4%).

### 3.2 Measures

This study used a five-point Likert scale ranging from 1 (*strongly disagree*) to 5 (*strongly agree*). To measure narcissistic leadership, we used a six-item scale developed by Hochwarter and Thompson (2012), which employees used to evaluate their leaders. A sample item is: “My leader exhibits a high degree of self-centeredness.”

Leaders evaluated their employees using a nine-item scale developed by Janssen (2000). A sample item is: “This employee mobilizes support for innovative ideas (idea promotion).”

Employees self-evaluated their resilience on a five-item scale developed by Al-Hawari et al. (2020). A sample item is: “I typically navigate challenges in some manner at work.”

Participants assessed supportive team climate using a five-item scale adapted from the Supportive Organizational Climate (SOC) Inventory developed by Luthans et al. (2008). The items capture employees’ shared perceptions of supportive and respectful interaction norms within their team. A sample item is: “My leader consistently treats everyone with respect,” which reflects a commonly experienced team-level climate of fairness and support rather than an individual leader–member exchange.

#### 3.2.1 Control variables (dummy variables)

This study employs dummy variables to represent categorical subgroups in the regression analyses, coding each category as 0 or 1.

At the individual level, we control for employees’ gender, age, educational level, and years of experience. We coded gender as male (0,0) and female (0,1). We categorize age as follows: 20–30 (0,0,0,0), 31–40 (0,1,0,0), 41–50 (0,0,1,0), and over 50 (0,0,0,1). We code educational attainment as junior college or below (0,0,0), bachelor’s degree (0,1,0), and master’s degree or above (0,0,1). We code years of experience as under 2 years (0,0,0,0), 2–5 years (0,1,0,0), 6–10 years (0,0,1,0), and over 10 years (0,0,0,1).

At the team level, we control for leaders’ gender, age, educational level, years of experience, and team size. We coded leaders’ gender as male (0,0) and female (0,1). We categorize leaders’ age as 20–30 (0,0,0,0), 31–40 (0,1,0,0), 41–50 (0,0,1,0), and over 50 (0,0,0,1). We code leaders’ education as junior college degree or below (0,0,0), bachelor’s degree (0,1,0), and master’s degree or above (0,0,1). We code leaders’ years of experience as under 2 years (0,0,0,0), 2–5 years (0,1,0,0), 6–10 years (0,0,1,0), and over 10 years (0,0,0,1). We categorize team size as 2–5 members (0,0,0), 6–9 members (0,1,0), and more than 10 members (0,0,1).

This coding approach allows us to incorporate categorical variables effectively into the regression models.

### 3.3 Data analysis

This study employs Mplus 8.3 to test the hypotheses with a multilevel model. To determine whether we should aggregate variables at the team level, we compute three indicators: the within-group agreement index (RWG), the intraclass correlation coefficient (ICC1), and the reliability of the group mean score (ICC2) (Bliese, 2000). For narcissistic leadership, ICC1 equals 0.600, indicating that 60.0% of the variance reflects between-group differences. ICC2 equals 0.900, which exceeds the 0.70 threshold and demonstrates acceptable reliability of the group mean. For the supportive team climate, ICC1 equals 0.660 and ICC2 equals 0.910, both of which indicate strong between-group variance and sufficient group-level reliability.

We also find RWG values of 0.885 for narcissistic leadership and 0.796 for supportive team climate, both above the 0.70 benchmark. These results indicate adequate within-group agreement and justify aggregating these constructs at the team level.

Based on these findings, we treat narcissistic leadership and supportive team climate as team-level variables, whereas we model employee resilience and employees’ innovative behaviors at the individual level.

We then conduct a null model test to determine whether multilevel modeling is necessary by examining how much variance in the dependent variables occurs at the group level. Table 1 shows that individual-level variance accounts for 54.9% of the variance in employee resilience and 50.8% of the variance in employees’ innovative behaviors. The ICC values for both outcomes exceed 0.138, which indicates a significant level of between-group heterogeneity (Snijders and Bosker, 2011). These findings confirm the appropriateness and necessity of using multilevel analyses.

**Table 1:** Null model test.

Statistic	Employee resilience	Employees’ innovative behavior
Intraclass correlation coefficient (ICC)	54.9%	50.8%

## 4 Data analysis and results

### 4.1 Preliminary analyses

To establish the discriminant validity of the study variables, we conducted a confirmatory factor analysis (CFA) using R at the individual level. The CFA focused on evaluating whether narcissistic leadership, employee resilience, supportive team climate, and employees’ innovative behavior represent empirically distinct constructs. As the primary purpose of this analysis was to assess measurement distinctiveness rather than cross-level variance, we estimated the CFA as a single-level measurement model and did not explicitly model team-level nesting. The results, presented in Table 2, indicate that the hypothesized four-factor model demonstrated an excellent fit to the data (*χ*^2^ = 247.497, df = 269, *χ*^2^/df = 0.920, CFI = 1.000, TLI = 1.000, RMSEA = 0.000, SRMR = 0.024), and substantially outperform alternative models. These findings provide strong evidence for the discriminant validity of the four focal constructs and support the adequacy of the measurement model for subsequent hypothesis testing.

**Table 2:** Results of confirmatory factor analysis.

Model	DF	CMIN	CMIN/DF	CFI	TLI	RMSEA	SRMR
Four-factor model (NL, ER, STC, EIB)	269	247.497	0.920	1.000	1.000	0.000	0.024
Three-factor model (NL, ER + STC, EIB)	272	1520.296	5.589	0.798	0.777	0.102	0.108
Two-factor model (NL + ER + STC, EIB)	274	2371.041	8.653	0.660	0.628	0.132	0.113
One-factor model (NL + EIB + ER + STC)	275	3200.712	11.639	0.526	0.483	0.155	0.133

NL, narcissistic leadership; EIB, employees’ innovative behaviors; ER, employee resilience; STC, supportive team climate; DF, degrees of freedom.

Because self-reported data may be subject to common method bias, we conduct Harman’s single-factor test (Harman, 1976) to assess the possible impact of common method variance. The results indicate that the first unrotated factor represents 35.17% of the total variance, which is below the commonly accepted threshold of 40%. Therefore, common method bias is not a significant issue in this study.

We also assessed multicollinearity because the regression models included multiple explanatory variables. All variance inflation factor (VIF) values fell below 5, indicating no multicollinearity and reinforcing the robustness of the regression results.

Table 3 presents the reliability and validity statistics for all measured constructs. This analysis reveals that the Cronbach’s alpha coefficients for all variables exceeded the recommended threshold of 0.70, demonstrating satisfactory internal consistency (George and Mallery, 2024). Additionally, all the Average Variance Extracted (AVE) values exceeded 0.50, while the Composite Reliability (CR) values exceeded 0.70, thereby adhering to the widely accepted standards. These results confirm that all constructs exhibit acceptable reliability and convergent validity, thus affirming their appropriateness for further hypothesis testing and structural analysis.

**Table 3:** Reliability and validity of scales.

Variable	Item	Cr-Alpha	Factor loading	CR	AVE
Narcissistic leadership	6	0.900	0.755–0.787	0.901	0.602
Employee resilience	5	0.906	0.798–0.819	0.906	0.658
Supportive team climate	5	0.908	0.821–0.843	0.908	0.664
Employees’ innovative behavior	9	0.901	0.675–0.793	0.902	0.506

Cr-alpha, Cronbach’s alpha; CR, composite reliability; AVE, average variance extracted.

Table 4 presents the averages, standard deviations (SD), and intercorrelations for all measured variables. The maximum absolute correlation coefficient is 0.422, which falls below the commonly accepted threshold of 0.50. This result shows that multicollinearity does not pose a concern in this dataset.

**Table 4:** Descriptive statistics and correlations.

Variable	Mean	SD	1	2	3	4	5	6	7	8	9	10	11	12	13	14
Individual level																
1. Gender (female)	0.510	0.500														
2. 31–40 years old	0.391	0.489	0.021													
3. 41–50 years old	0.335	0.472	−0.010	−0.569***												
4. Over 50 years old	0.164	0.370	−0.003	−0.355***	−0.314***											
5. Bachelor’s degree	0.618	0.486	0.025	0.042	0.166***	−0.189***										
6. Master’s degree	0.196	0.397	−0.061	−0.081	−0.134**	0.164***	−0.627***									
7. 2–5 years	0.184	0.388	0.072	0.356***	−0.337***	−0.211***	0.004	−0.015								
8. 6–10 years	0.364	0.482	−0.015	0.025	0.215***	−0.335***	0.066	−0.043	−0.360***							
9. More than 10 years	0.432	0.496	−0.009	−0.270***	0.084	0.509***	−0.062	0.404	−0.414***	−0.659***						
10. Employee resilience	3.387	1.080	−0.070	0.040	−0.076	0.040	−0.200***	0.095*	0.017	−0.008	−0.006	(0.811)				
11. EIB	3.585	0.841	0.006	0.000	−0.017	0.099*	−0.161***	0.116*	−0.037	−0.014	0.045	0.422***	(0.711)			
Team level																
1. Gender (female)	0.369	0.485														
2. 31–40 years old	0.298	0.460	0.042													
3. 41–50 years old	0.488	0.503	−0.105	−0.636***												
4. Over 50 years old	0.167	0.375	0.055	−0.219**	−0.437***											
5. Bachelor’s degree	0.702	0.460	0.012	0.139	−0.146	−0.058										
6. Master’s degree	0.250	0.436	0.014	−0.135	0.151	0.037	−0.887***									
7. 2–5 years	0.179	0.385	−0.035	−0.444**	−0.455***	−0.209	0.100	−0.054								
8. 6–10 years	0.167	0.375	−0.210	−0.221*	0.394***	−0.200	0.012	0.037	−0.209							
9. More than 10 years	0.655	0.478	0.192	−0.185	0.058	0.325***	−0.089	0.014	−0.642***	−0.616***						
10. 6–7 members	0.833	0.278	0.037	−0.008	0.136	−0.135	−0.086	0.124	−0.141	0.212	−0.053					
11. 8–10 members	0.048	0.214	−0.171	−0.146	0.117	0.050	−0.099	0.129	−0.104	0.200	−0.073	−0.067				
12. More than 10 members	0.024	0.153	0.204	0.069	0.004	−0.070	−0.240*	0.271*	−0.073	−0.070	0.133	−0.047	−0.035			
13. Narcissistic leadership	3.248	0.890	0.077	0.154	−0.094	−0.076	0.132	−0.022	0.062	0.061	−0.097	−0.060	−0.229*	0.087	(0.776)	
14. Supportive team climate	3.252	0.792	−0.086	0.060	0.039	−0.068	−0.057	−0.026	0.001	−0.084	0.065	−0.030	−0.031	−0.061	−0.397***	(0.815)

EIB, employees’ innovative behavior. The square root of AVE is along the diagonal. *N* employees = 445, *N* leaders = 84; * *p* < 0.05, ** *p* < 0.01, *** *p* < 0.001.

We assess discriminant validity by comparing the square root of each construct’s average variance extracted (AVE) with its correlations with other constructs. All constructs meet this criterion, which supports satisfactory discriminant validity.

### 4.2 Hypothesis tests

To test the proposed multilevel mediation and moderation hypotheses, we use multilevel structural equation modeling (MSEM) following Preacher et al. (2016). Given that employees were within teams, we explicitly separated within- and between-level variance components in the MSEM framework. We modeled narcissistic leadership and supportive team climate at the team level, whereas we specified employee resilience and employees’ innovative behaviors at the individual level, forming a 2-1-1 mediation model.

Before analysis, we group-mean-centered individual-level predictors and grand-mean-centered team-level variables in line with established cross-level modeling practices. We constructed interaction terms involving narcissistic leadership and supportive team climate at the between-level to test the hypothesized moderating effects. We handled missing data using full information maximum likelihood (FIML) and estimated indirect and conditional indirect effects based on model-implied path coefficients and their confidence intervals within the MSEM framework.

Table 5 shows that narcissistic leadership at the team level exerted a significant negative direct effect on employees’ innovative behaviors at the individual level, supporting Hypothesis 1 (*β* = −0.492, *p* < 0.001, 95%CI [−0.657, −0,309]). Narcissistic leadership (NL) also showed a significant negative association with employee resilience at the individual level, supporting *Hypothesis 2* (*β* = −0,557, *p* < 0.001, 95%CI [−0.749, −0.365]). Employee resilience (ER), in turn, had a significant positive effect on employees’ innovative behaviors, supporting *Hypothesis 3* (*β* = 0.200, *p* < 0.001, 95%CI [0.110, 0.291]). Mediation analysis further showed that employee resilience transmitted a significant indirect negative effect of narcissistic leadership on employees’ innovative behaviors (EIB), supporting *Hypothesis 4* (*β* = −0.112, *p* < 0.01, 95%CI [−0.175, −0.048], NL → ER → EIB).

**Table 5:** Results of multilevel structural equation modeling (MSEM).

Path	Estimates	S.E.	T-value	95% CI	*p*-value	Remarks
Direct effect						
NL → EIB	−0.492***	0.093	−5.270	[−0.657, −0,309]	0.000	Supported (*H1*)
NL → ER	−0.557***	0.098	−5.684	[−0.749, −0.365]	0.000	Supported (*H2*)
ER → EIB	0.200***	0.046	4.329	[0.110, 0.291]	0.000	Supported (*H3*)
Mediating effect						
NL → ER → EGIB	−0.112**	0.033	−3.437	[−0.175, −0.048]	0.001	Supported (*H4*)
Moderating effect						
NL × STC → EIB	0.337***	0.096	3.525	[0.150, 0.525]	0.000	Supported (*H5*)
NL × STC → ER	0.318**	0.120	2.653	[0.083, 0.554]	0.008	Supported (*H6*)

NL, narcissistic leadership; EIB, employees’ innovative behavior; ER, employee resilience; STC, supportive team climate; * *p* < 0.05, **p < 0.01, ***p < 0.001.

**Figure 2:**
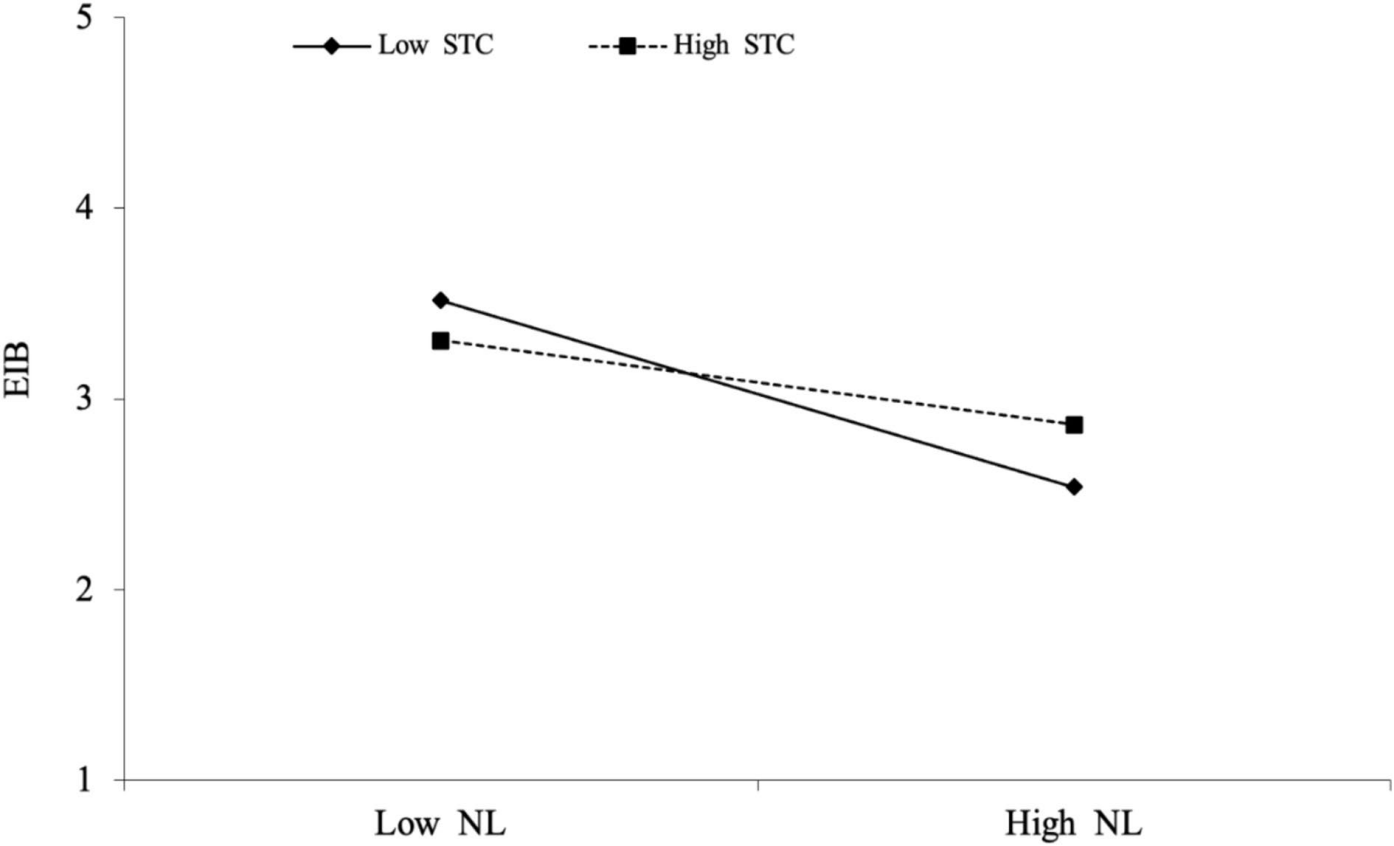
Moderating effect diagram of supportive team climate on narcissistic leadership–employees’ innovative behaviors.

To test moderation, we examined how supportive team climate influenced the relationship between narcissistic leadership and employees’ innovative behaviors. The results indicate that a supportive team climate significantly weakened the negative relationship between narcissistic leadership and employees’ innovative behaviors when support was high rather than low. The interaction effect was significant (*β* = 0.337, *p* < 0.001, 95% CI [0.150, 0.525]), indicating that a supportive team climate (STC) mitigates the detrimental effect of narcissistic leadership on innovation. These findings support *Hypothesis 5* (NL × STC → EIB). Figure 2 presents the moderating effect of a supportive team climate on narcissistic leadership and employees’ innovative behaviors.

**Figure 3:**
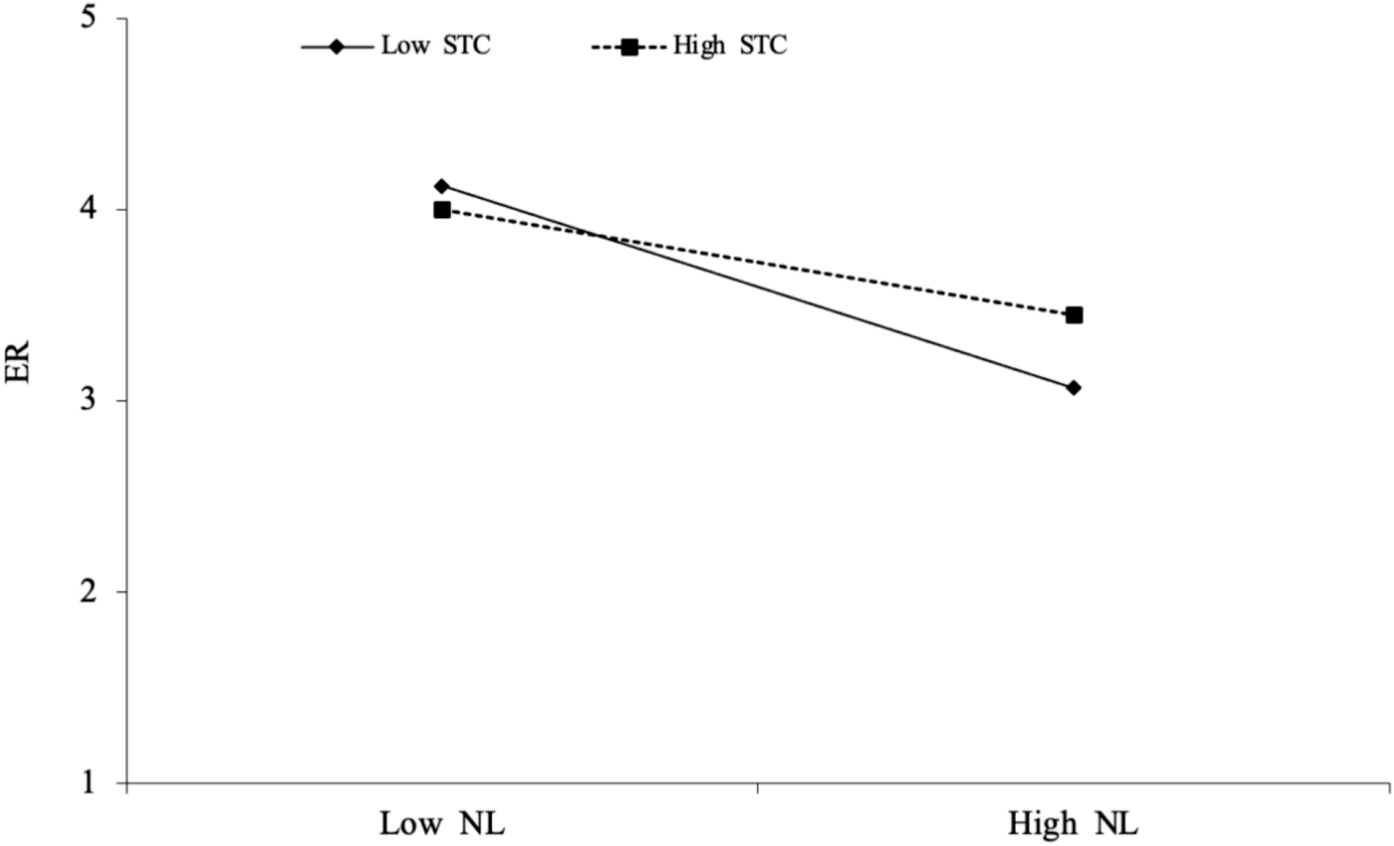
Moderating effect diagram of supportive team climate on narcissistic leadership–employee resilience.

We also tested whether supportive team climate moderates the relationship between narcissistic leadership and employee resilience. The interaction term (NL × STC) was significant (*β* = 0.318, *p* < 0.01, 95% CI [0.083, 0.554]), indicating that a supportive team climate significantly reduces the negative impact of narcissistic leadership on employee resilience. In other words, supportive teams buffer the negative effects of narcissistic leadership by helping employees maintain psychological adaptability. These results support *Hypothesis 6* and highlight the protective role of team climate in sustaining employee resilience under challenging leadership conditions. Figure 3 illustrates the moderating effect of a supportive team climate on the relationship between narcissistic leadership and employee resilience.

**Figure 4:**
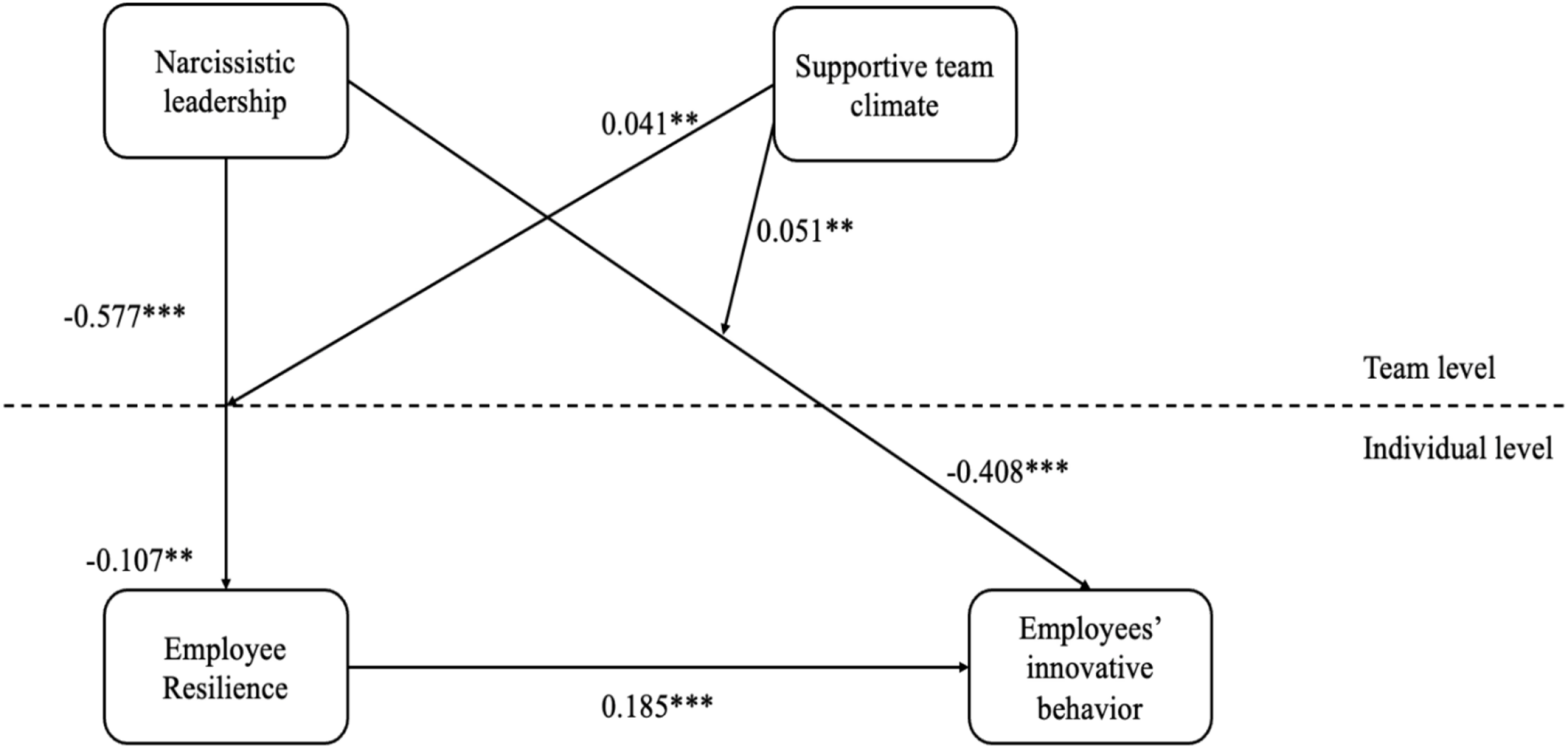
Robustness test results.

To further assess the robustness of these findings, we conducted an additional analysis in R using an alternative analytical approach. Although we tested the primary hypotheses using MSEM in Mplus, the robustness analysis re-estimated the key paths using a different estimation framework to examine whether the direction and significance of the results remain consistent. As shown in Figure 4, the results of the robustness analysis closely mirror the main MSEM findings, which further support the stability and reliability of the proposed model.

## 5 Discussion

This study examines the multilevel mechanisms through which narcissistic leadership shapes employees’ innovative behaviors in technology-oriented SMEs, with a particular focus on the mediating role of employee resilience and the moderating role of supportive team climate. By integrating individual psychological resources and team-level contextual factors, the findings provide a more nuanced understanding of how dark-side leadership influences employee innovation.

First, the results show that narcissistic leadership negatively affects employees’ innovative behaviors. Leadership characterized by self-centeredness, excessive control, and emotional neglect may constrain employees’ willingness and capacity to engage in innovation. Consistent with prior research on destructive leadership, narcissistic leaders appear to undermine employees’ autonomy and psychological safety, which are essential conditions for sustained innovative engagement.

Second, employee resilience plays a significant mediating role in the relationship between narcissistic leadership and innovative behavior. Specifically, narcissistic leadership reduces employee resilience, which in turn lowers employees’ innovative behaviors. This finding highlights resilience as a critical psychological mechanism by linking negative leadership to employee outcomes. Employees who experience sustained psychological strain and resource depletion under narcissistic leadership may find it increasingly difficult to recover, adapt, and invest effort in innovation-related activities.

Third, the results demonstrate that supportive team climate moderates the negative effects of narcissistic leadership on employee resilience and innovative behavior. In highly supportive team environments, supportive climates significantly weaken the adverse impact of narcissistic leadership. This finding suggests that team-level social and emotional resources compensate for deficiencies in leadership behavior, enable employees to regulate negative emotions, maintain psychological balance, and remain engaged in innovative work despite exposure to self-serving or controlling leaders.

Taken together, these findings underscore the importance of considering individual psychological resources and team-level contextual factors when examining the consequences of narcissistic leadership. Although narcissistic leadership poses substantial risks to employee resilience and innovation, supportive team climates and resilient employees can meaningfully buffer these negative effects, thereby shaping how leadership behavior translates into employee innovation outcomes.

### 5.1 Theoretical implications

This study enhances the literature on destructive leadership and organizational behavior by offering an in-depth analysis of how narcissistic leadership influences employees’ innovative behaviors. Rather than merely reiterating that narcissistic leadership harms employee outcomes, this study advances leadership research by demonstrating how such negative effects unfold and under what conditions organizations can constrain them. Building on prior studies (Chatterjee and Hambrick, 2007; Wang et al., 2022), this study empirically confirms that narcissistic leadership negatively influences employee innovation by undermining the psychological resources that support sustained creativity and experimentation. By situating this relationship within the context of technology-oriented SMEs, this study extends recent discussions on the dark side of leadership in innovation-driven environments (Fang et al., 2024; Khoso et al., 2025) and highlights the heightened vulnerability of employees in resource-constrained organizational settings.

Second, this study advances understanding of the psychological mechanisms underlying destructive leadership by identifying employee resilience as a key mediating process. Utilizing COR theory (Hobfoll, 1989; Kiazad et al., 2014), this research conceptualized employee resilience as a dynamic and developable psychological resource that narcissistic leadership can deplete over time, rather than as a stable individual trait. In doing so, the study extends recent resilience research by positioning resilience as a central explanatory mechanism linking destructive leadership to innovation outcomes (Mohammad et al., 2024; Srivastava et al., 2025). This perspective clarifies why innovation declines under narcissistic leadership: employees lose adaptive capacity and recovery resources, not simply motivation or ability.

Third, this study makes a distinct multilevel theoretical contribution by highlighting the buffering role of supportive team climate. The findings show that team-level contextual resources can attenuate the negative effects of narcissistic leadership on employee resilience and innovative behavior. These findings challenge a predominantly leader-centric view of management and demonstrate that leadership effects operate within collective team contexts. Supportive team climates provide salient social cues and shared resources that reshape how employees interpret and respond to destructive leadership, answering calls for multilevel investigations of dark-side leadership (Azam and Rizvi, 2021; Zhang et al., 2025).

Finally, this study contributes methodologically by adopting a multilevel and time-lagged design, which enables a more rigorous examination of cross-level relationships between leadership behavior, team climate, and individual innovation outcomes. By integrating individual psychological processes with team-level contextual conditions in Chinese technology-oriented SMEs, this research offers a more context-sensitive account of how destructive leadership shapes innovation over time.

### 5.2 Managerial implications

This study provides several concrete managerial implications for organizations, particularly SMEs operating in innovation-driven and resource-constrained environments.

First, given the consistent negative effect of narcissistic leadership on employees’ innovative behaviors, organizations should focus on identifying destructive leadership tendencies and limit the extent to which such tendencies shape innovation-related processes. In SMEs, where replacing leaders often proves difficult, organizations can establish clear role boundaries and formalized decision procedures to reduce excessive leader discretion in areas critical to innovation, such as idea evaluation, resource allocation, and credit attribution (Braun, 2017; Norouzinik et al., 2022). By constraining unilateral control and increasing transparency in innovation-related decisions, organizations can prevent narcissistic leadership styles from becoming deeply embedded in everyday innovation practices.

Second, the mediating role of employee resilience underscores the importance of strengthening employees’ psychological resources as a practical managerial intervention. Rather than relying solely on resource-intensive formal training programs, organizations can embed resilience-supportive practices into everyday work design and routines. For example, managers can provide employees greater task autonomy, adjust workloads realistically, and create opportunities for recovery following periods of high strain, thereby facilitating continuous resource replenishment (Luthans et al., 2006; Sutcliffe and Vogus, 2003). Such embedded practices help employees sustain adaptive functioning and innovative engagement, particularly when leadership support remains limited or inconsistent.

Third, managers can actively cultivate a supportive team climate to buffer the negative consequences of narcissistic leadership. In practice, this involves shaping team-level interaction patterns so that support, feedback, and recognition do not depend solely on the leader. Managers can, for instance, institutionalize regular team reflection meetings, organize structured peer problem-solving sessions, and implement peer-based recognition mechanisms to encourage mutual respect and collective engagement (Nembhard and Edmondson, 2006; Zhou et al., 2019). Through these team-based practices, employees gain access to alternative social and emotional resources within the group, which helps protect employee resilience and sustain innovative behavior even when leadership support is weak or inconsistent.

Overall, this study suggests that organizations cannot rely solely on correcting leader behavior to protect innovation. Instead, a dual strategy, combining the management of destructive leadership risks with the deliberate cultivation of employee resilience and supportive team climates, offers a more feasible and sustainable approach to maintaining innovative performance in SMEs.

### 5.3 Limitations and future research directions

This study makes significant theoretical and practical contributions; however, several limitations suggest directions for future research.

First, the study drew data from SMEs located in a specific Chinese region, which potentially limits generalizability. To enhance external validity, future research should expand the sampling frame to encompass organizations from various industries and national contexts.

Second, although the study used a time-lagged design and supervisor-rated innovative behavior to mitigate common method bias, it measured narcissistic leadership and supportive team climate from the same employees at the same time. As a result, shared perceptual biases might influence the observed relationships. Future studies can reduce this concern by separating these measures temporally or incorporating multi-source assessments to strengthen methodological robustness.

Third, this study focused exclusively on employee resilience as a mediating variable. Since leadership effects often involve multiple psychological processes, future research should incorporate additional mediators such as psychological safety, emotional exhaustion, or work engagement to build more comprehensive models.

Fourth, this study conceptualized narcissistic leadership predominantly as a negative and maladaptive influence. However, recent research indicates that narcissistic traits may exhibit nonlinear effects, such as U-shaped relationships, with moderate levels yielding positive outcomes in certain contexts, such as innovation-driven environments. Future studies should explore such nonlinear patterns or differentiate between adaptive and maladaptive narcissism to understand its complex impact on resilience and innovation behavior.

Finally, although this study examined supportive team climate as a moderating variable, it did not explore the antecedents of such a climate. Future studies should investigate the specific managerial practices or leadership behaviors that foster supportive team climates and enhance their buffering effect against destructive leadership.

## 6 Conclusion

This study developed an integrated multilevel framework explaining how narcissistic leadership constrains employees’ innovative behaviors in SMEs. The findings indicate that narcissistic leadership influences innovation indirectly by depleting employees’ psychological resources. Employee resilience serves as a central mediating mechanism, whereas supportive team climate shapes the strength of this process.

By grounding these mechanisms in the COR and self-determination theories, this research advances a resource-based explanation of dark-side leadership in innovation contexts. The framework highlights the importance of fostering employee resilience and supportive team environments to sustain innovation when organizations face adverse or self-centered leadership.

## Data Availability

The raw data supporting the conclusions of this article will be made available by the authors, without undue reservation.
